# A trial-ready cohort for finding unmet needs and improving quality of life among patients with esophageal cancer: a multicenter prospective cohort study

**DOI:** 10.3389/fonc.2025.1607741

**Published:** 2025-07-23

**Authors:** Jiyoon Han, Genehee Lee, Juhee Cho, Somin You, Young Mog Shim, Yong Soo Choi, Jong Ho Cho, Seong Yong Park, Yeong Jeong Jeon, Junghee Lee, Danbee Kang, Hong Kwan Kim

**Affiliations:** ^1^ Department of Clinical Research Design and Evaluation, Samsung Advanced Institute for Health Sciences & Technology, Sungkyunkwan University, Seoul, Republic of Korea; ^2^ Center for Clinical Epidemiology, Samsung Medical Center, Seoul, Republic of Korea; ^3^ Patient-Centered Outcomes Research Institute, Samsung Medical Center, Seoul, Republic of Korea; ^4^ Departments of Health, Behavior, and Society and Epidemiology, Johns Hopkins Bloomberg School of Public Health, Baltimore, MD, United States; ^5^ Department of Thoracic and Cardiovascular Surgery, Samsung Medical Center, Sungkyunkwan University School of Medicine, Seoul, Republic of Korea

**Keywords:** esophageal cancer survivor, health-related quality of life, trial-ready cohort, cancer survivorship, long-term follow-up, patient-reported outcome

## Abstract

Although esophageal cancer survivors experience lower health-related quality of life (HRQoL), it is hard to provide proper supportive care due to difficulties to find potential target population. This study aims to develop a trial-ready cohort (TRC) to assess the unmet needs and HRQoL of survivors of esophageal cancer. This prospective, multicenter TRC study will include 600 patients diagnosed with primary esophageal cancer who have undergone curative treatment. Exclusion criteria include the presence of synchronous malignancies, severe cognitive impairment, and residence outside of Korea. Patients are recruited from both existing cohort studies and newly diagnosed cases beginning in June 2023. At the time of enrollment into the TRC, participants provide informed consent, including agreement to be contacted and considered for relevant clinical trials when suitable interventions become available. Data is collected across five domains: sociodemographic characteristics, health behaviors, disease and treatment information, nutritional status, and quality of life. Study visits are scheduled at diagnosis, prior to surgery, at 1, 3, 6, and 12 months post-surgery, and annually thereafter for up to 10 years. As of June 2025, a total of 448 participants have been enrolled, representing approximately 75% of the target. To achieve this, our TRC employs three key strategies. First, we leverage three existing prospective studies to efficiently identify and enroll long-term survivors. Second, we collect a wide range of data on HRQoL, health behaviors, and environmental factors to enable a multidimensional understanding of survivorship. Third, we collect multiple times within short-term interval points, allowing seamless linkage between the cohort and appropriate trials. This study effectively designs a trial-ready cohort of survivors of esophageal cancer using a unique strategy to overcome cohort construction challenges, aiming to generate valuable data on quality of life and serve as a platform for tailored interventions.

## 1 Introduction

Esophageal cancer is the eleventh most common cancer worldwide and seventh in terms of mortality (1). Over the past 20 years, the five-year survival rate of esophageal cancer increased from 21.7% (2001–2005) to 43.2% (2018–2022). Consequently, the number of esophageal cancer survivors has steadily increased (2). However, most previous studies have followed esophageal cancer survivors for only 1–2 years postoperatively (3–5) or were cross-sectional in design, limiting insights into the long-term impact of treatment (6–8). Only a few cohort studies have extended follow-up beyond five years, but they included fewer than 200 patients, with high rates of loss to follow-up (9, 10). Although some previous studies have tracked esophageal cancer survivors’ QoL for over 5 years postoperatively, they exhibit limitations. A study in China reported follow-up data up to 9 years after surgery for over 300 patients, however, it cannot be regarded as a true longitudinal assessment, as more than 60% of participants completed the QoL assessment only once (11). Similarly, another Chinese study tracked 232 postoperative patients for a median of 80 months, yet QoL data were collected only during the first two years, with no assessments beyond 24 months (12). The OSCAR study from Sweden followed over 400 patients and achieved repeated fatigue assessments up to 5 years with relatively high retention. However, its scope was limited to cancer-related fatigue, lacking a broader multidimensional evaluation of QoL (13).

Based on the previous studies, it is well established that esophageal cancer survivors frequently experience substantially impaired health-related quality of life (HRQoL), primarily due to slow postoperative recovery and persistent symptoms (14). More than 50% of patients who had undergone esophagectomy reported dysphagia, 60% experienced dumping syndrome, and 33% suffered from severe weight loss (>10%) (15). While cancer survivorship guidelines recommend comprehensive follow-up care (16), current practices remain largely limited to medical surveillance and tumor-specific monitoring. In particular, esophageal cancer survivors often face socioeconomic disadvantages and have limited access to recruitment platforms (17), making it difficult to identify eligible individuals for supportive care interventions and to ensure timely delivery of appropriate care (18).

Given these barriers to long-term follow-up and interventional research, trial-ready cohorts (TRCs) represent a promising strategy. TRCs are prospective, well-characterized cohorts that enable the continuous identification of high-risk subgroups and allow for the rapid initiation of targeted intervention trials (19). Although TRCs have been successfully adopted in various fields, including cardiology and rare cancers, their application to cancer survivorship remains limited. Therefore, the objective of this study is to describe the protocol for the development and implementation of a prospective, platform-based, trial-ready cohort.

## 2 Methods

### 2.1 Study design and study population

**Figure 1:**
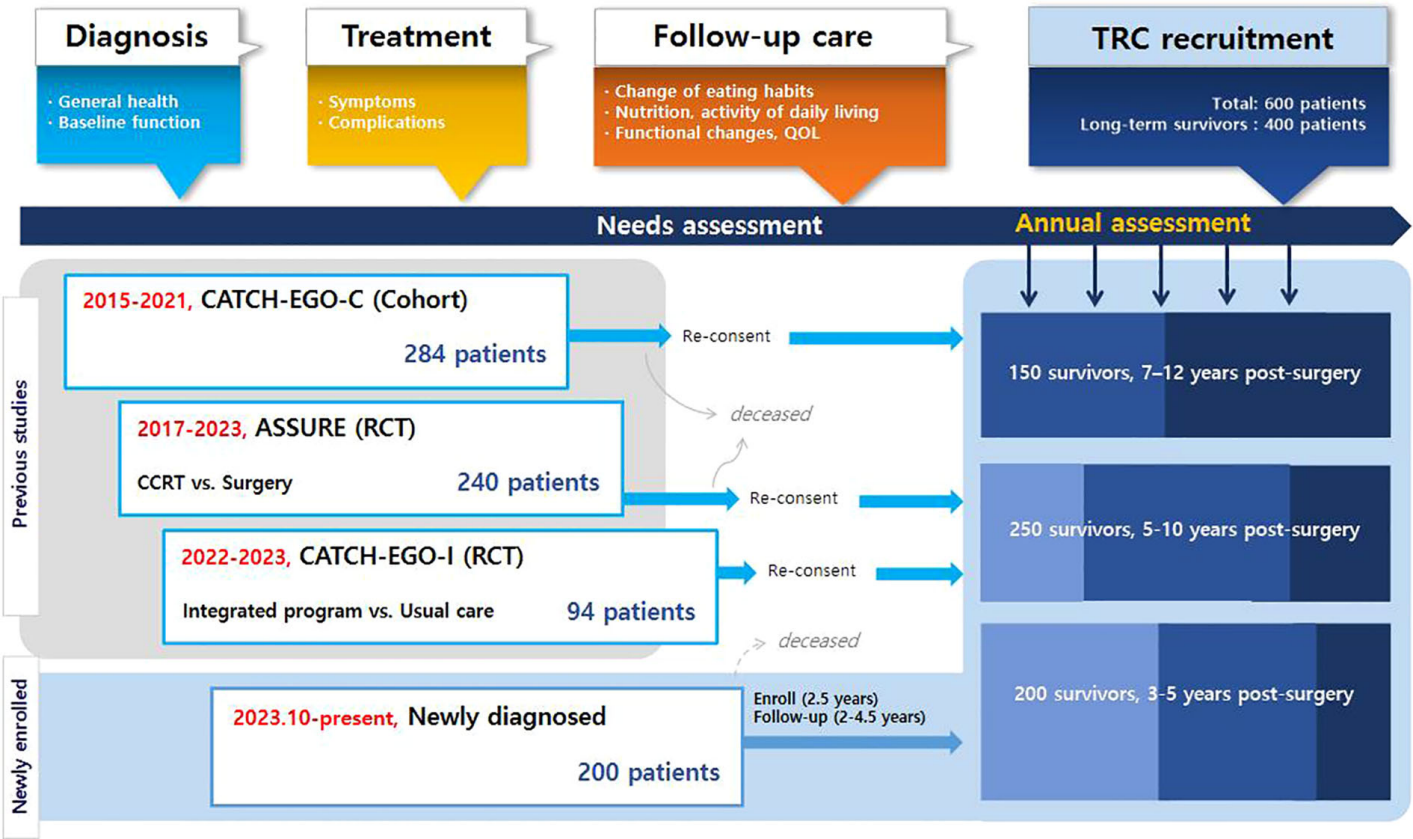
Diagram of the cohort design.

This is a prospective multicenter TRC study involving patients with esophageal cancer (**Figure 1**). The eligibility criteria are as follows: 1) patients diagnosed with primary esophageal cancer, including squamous cell carcinoma or adenocarcinoma, confirmed through endoscopic evaluation, imaging studies such as CT or PET-CT, and histopathological examination of biopsy specimens by board-certified pathologists, 2) patients who had or will have treatment for curative purposes, 3) patients who understand and write Korean at a native level, and 4) patients who provided informed consent. Patients are excluded if 1) they have a previous history at the time of esophageal cancer diagnosis including synchronous cancers, other cancers within 3 years, or esophagectomy, 2) they have severe cognitive impairment, including dementia and schizophrenia or 3) reside overseas that hinders regular participation. At the time of enrollment into the TRC, participants provide informed consent, including agreement to be contacted and considered for relevant clinical trials when suitable interventions become available.

Given the low incidence and relatively high mortality rates, we recruit patients from three existing cohort studies of esophageal cancer as well as from newly diagnosed cases. For newly diagnosed patients, no restrictions are applied regarding age, comorbidities, or treatment modality in order to capture a broader range of survivorship needs.

Starting in June 2023, patients participating in the existing cohorts are recruited during their surveillance visits to treatment hospitals, typically during annual visits to outpatient clinics Detailed information on existing cohorts are followings: The CATCH-EGO-C study (ClinicalTrials.gov ID: NCT03231462) is a prospective cohort study investigating how perioperative physical activity affects postoperative pulmonary complications and quality of life (QoL) in patients with esophageal cancer. The study recruited 284 patients with stage I-II esophageal cancer who underwent curative surgery at Samsung Medical Center since Mar 2015. The ASSURE study (ClinicalTrials.gov ID: NCT03306901) is a multicenter randomized controlled non-inferiority trial that compared the survival rates of chemoradiotherapy and esophagectomy after endoscopic submucosal dissection (ESD) in patients with superficial esophageal squamous cell carcinoma (clinical stage T1N0M0). The trial enrolled 240 patients (121 and 119 in the surgery and concomitant chemoradiotherapy groups, respectively) in stage I starting in Oct 2017, and is following up on clinical outcomes from seven hospitals across South Korea (Samsung Medical Center, Asan Medical Center, Seoul National University Hospital, Seoul National University Bundang Hospital, National Cancer Center, Gangnam Severance Hospital, and Pusan National University Hospital). Another study, CATCH-EGO-I (CRIS Registration Number: KCT0006446), is a randomized controlled trial that is comparing severe weight loss between patients with stage I-IV esophageal cancer receiving weekly integrated program for 2 months (n=48) and usual care (n=46) after surgery. The program consisted of delivery of oral nutritional supplements (ONS) and protein powder, and exercise counselling based on individual requirement and lifelog data, following up to 12 months (**Table 1**) At enrollment, the three cohorts differed in prior follow-up duration (CATCH-EGO-C: 8.3 years, ASSURE: 5.7 years, and CATCH-EGO-I: 1.2 years), resulting in participants being enrolled at different stages of follow-up depending on trial timelines. These variations were addressed during data harmonization by aligning common variables and timepoints.

**Table 1:** Description of existing esophageal cancer cohorts.

Study name	Impact of perioperative physical activity on postoperative pulmonary complications and quality of life among esophageal cancer patients: A prospective cohort study	A randomized multicenter noninferiority trial comparing chemoradiotherapy versus esophagectomy after endoscopic submucosal dissection for superficial esophageal squamous cell carcinoma	Application of lifelog-based physical activity and nutritional intervention for minimizing postoperative weight-loss among patients with esophageal cancer
Study nickname	CATCH-EGO-C	ASSURE	CATCH-EGO-I
Study design/Center	Prospective single center cohort	Multicenter randomized controlled trial	Single center randomized controlled trial
Inclusion Criteria	Patients who: 1. Are diagnosed with primary esophageal cancer with clinical stage I or II and are scheduled for curative esophagectomy and reconstruction surgery as primary treatment. 2. Are able to walk independently and understand the questionnaires. 3. Provided written informed consent. 4. Have no history of psychiatric disease requiring treatment.	Patients who: Are histologically diagnosed with squamous cell carcinoma of the esophagus, and age ≥ 19 years and < 80 years. Are evaluated as clinical stage T1N0M0 according to upper GI endoscopy, endoscopic ultrasound, and chest computed tomography scans. Are confirmed with submucosal invasion (pathologic T1b) or lympho-vascular invasion through endoscopic submucosal dissection(ESD). Have undergone complete resection via ESD, with at least one lesion showing pathologic submucosal invasion (pT1b) or lympho-vascular invasion, in the case of patients with multiple lesions. Have adequate hematologic function, defined by an absolute neutrophil count (ANC) ≥1000/µL, hemoglobin ≥8 g/dL, and platelets ≥85,000/µL. Have adequate hepatic and renal function, defined by aspartate transaminase (AST) and alanine transaminase (ALT) ≤ 2.5 times the upper limit of normal; total bilirubin ≤1.5 times the upper limit of normal; and creatinine clearance ≥30 mL/min/1.73m^2^. Provided written informed consent.	Patients who: Are diagnosed with esophageal cancer with clinical stage I-IV and are scheduled for esophagectomy, and age ≥ 19 years and < 80 years. Are capable of independent walking and performing daily activities. Are evaluated with an ECOG performance status of 0-1. Are capable of using a smartphone. Provided written informed consent.
Exclusion Criteria	Patients who: Have underwent neoadjuvant treatment. Are confirmed to have a multiple cancers or history of another cancer within the last 3 years. Are not able to regularly participate due to oversea residency.	Patients who: Are diagnosed with cervical esophageal cancer (proximal to 20 cm from the incisor teeth). Are suspected or confirmed with regional lymph node metastasis (cN+) or distant metastasis (cM1) on chest CT scans or positron emission tomography (PET)/CT scans [equivocal results are regarded as no metastasis; biopsy is acceptable if necessary (optional)]. Are diagnosed with recurrent esophageal cancer. Are confirmed with uncontrolled systemic disease, such as congestive heart failure, interstitial lung disease, severe pulmonary emphysema, or chronic renal failure, which makes the patient unsuitable for additional treatments (esophagectomy or concurrent chemoradiotherapy). Are not suitable for esophageal reconstruction using the gastric conduit (e.g., due to a previous history of gastrectomy). Are diagnosed with synchronous or metachronous multiple cancers within the past 3 years, except for skin cancer, well-differentiated thyroid cancer, carcinoma *in situ*, early cancer with curative endoscopic resection, or low-grade prostate cancer (Gleason score ≤6).	Patients who: Have difficulties in communication or are undergoing psychiatric treatment. Have a history of esophagectomy and reconstruction surgery (ESD allowed). Have a history of other cancer treatments within the past 3 years. Are not able to regularly participate due to oversea residency. Have concern of worsening of underlying condition during intervention: - Cardiac failure (New York Heart Association functional classes III and IV) or other cardiovascular diseases, where worsening of the condition is expected during moderate to high-intensity physical activity. - Inability to walk due to joint problems, paralysis, etc. - Impaired kidney function for whom protein intake is not recommended (estimated GFR <60 mL/min/1.73m^2^). - Problems with oral intake after surgery, where the researcher determines participation is inappropriate for the intervention study. - Oral intake has not started within 14 days after surgery.
Intervention	Not applicable	Concurrent chemoradiotherapy vs. Esophagectomy	Integrated program including nutrition and exercise: Weekly delivery of oral nutritional supplements (ONS) and protein powder based on individual requirement, and exercise counselling based on lifelog data vs. Usual care
Primary Outcome	Postoperative pulmonary complications (pneumonia and atelectasis)	3-year overall survival	Incidence rate of significant weight loss in 2 months post-surgery (more than 10% reduction from preoperative weight)
Follow-up period	5 years	10 years	1 year
Follow-up timepoint	Baseline(Pre-surgery), immediately after surgery, 4, 8, 24, and 48 weeks post-surgery, and annually thereafter	Baseline(Pre-treatment), 3, 6, 12, 18, 24, 30, 36, 48, 60, 72, 84, 96, 108, and 120 months after the initiation of treatment in both arms	Baseline(Pre-surgery), discharge (if oral intake is possible), 1, 2, 6, 12 months post-surgery
Number of enrollments	284	240	94

Newly diagnosed patients are those with primary esophageal cancer planned for curative resection and reconstruction at clinical stages I-III, including those who receive neoadjuvant treatment. All patients are enrolled before the start of any treatment and recruited from the thoracic surgery outpatient clinics at the Samsung Medical Center, Asan Medical Center, Seoul National University Hospital, and Seoul National University Bundang Hospital. The physician explains the study to eligible patients and obtain their informed consent. To better understand the diverse unmet needs of patients, we have decided to expand the inclusion criteria to encompass patients with advanced stages of the disease, thereby capturing a broader range of experiences and challenges. Patients can be excluded from the study after enrollment under the following conditions: 1) enrolled in the study with the intention of receiving curative treatment (surgery or anticancer radiation therapy) for esophageal cancer but did not undergo treatment, or 2) primary treatment strategy shifted from curative to non-curative due to disease progression or other reasons. This study has been approved by the institutional review boards of all the participating hospitals.

### 2.2 Variables and measurement

Although the three source cohorts differ in study design, comprising observational and interventional studies, the data collection process has been standardized across all cohorts. This includes harmonization of variable definitions, measurement timepoints, and patient-reported outcome measures (PROMs), allowing for consistent and comparable data across participants regardless of cohort origin.

Information is collected on five domains: sociodemographics, health behavior, disease and treatment information, nutritional status, and QoL. Data are collected during regular clinic visits of the patients, as the visit schedules aligned as much as possible with the standard esophageal cancer treatment process. Visits are scheduled at the time of enrollment (baseline, T0); pre-surgery (T1); 1 (T2), 3 (T3), 6 (T4), and 12 months post-surgery (T5); and annually thereafter (T6–T19). T0 visit is planned at the time of diagnosis (pretreatment). The T1 visit applies only to patients who undergo neoadjuvant (radiation) therapy before surgery. The visit window for each timepoint varies: the T0 visit can be scheduled at any time between enrollment and treatment initiation, the T1 visit can be scheduled from 2 weeks after the completion of neoadjuvant treatment until before surgery, and the visit windows for T2, T3, T4, and T5 to T19 are ±14 days, -1 month, ± 1 month, and ±2 months, respectively.

The sociodemographic domain includes participants’ sex, date of birth, education level, marital status, occupation, and family income. Sociodemographic information is gathered using self-report questionnaires. Marital status, occupation, and family income which are expected to change over time, are collected at T0 and consecutively starting from T4, whereas all other data are collected only at T0.

The health behavior domain includes smoking and drinking status, alcohol addiction, eating habits, diet, physical activity, and care environment which provides information on the current state of the patient’s caregiver. This domain measures patients’ health-related behaviors and environmental factors that affect their behavioral choices. Data are collected at every study visit from T0, except for smoking and drinking status, alcohol addiction, physical activity, and care environment which are not evaluated at immediate post-surgery such as T2 and T3. Most of the variables use patient-reported outcome measurements (PROMs) for data collection (**Table 2**) except smoking and drinking status, eating problem, and care environment, which are assessed using self-developed questionnaires.

**Table 2:** Data collection for each domain.

Domain	Variable	Data collection method
Socio-demographic	Sociodemographic information	Self-developed questionnaire
Health behavior	Smoking and drinking status	Self-developed questionnaire
	Alcohol addiction	AUDIT^a^
	Dietary habit	MDA^b^
	Eating problem	Self-developed questionnaire
	Physical activity	IPAQ-SF^c^
	Care environment	Self-developed questionnaire
Disease and treatment information	Baseline comorbidity	Anatomic classification of diseases, EMR^d^
	Baseline pulmonary function	EMR^d^
	Esophageal cancer information	EMR^d^
	Cancer treatment information	EMR^d^
	Complications	STS & ESTS definition^e^, C-D classification^f^
	Disease progression	EMR^d^
	Survival status	EMR^d^, National institution
Nutritional status	Nutritional index	Laboratory test, PG-SGA-SF^g^, EMR^d^
	Muscle strength	Grip strength dynamometer
Quality of Life	Overall quality of life	EQ-5D-5L^h^
	Quality of life in cancer patient	EORTC QLQ-C30^i^
	Quality of life of patients with esophageal cancer	EORTC QLQ-OES18^j^
	Gastrointestinal symptoms	GSRS^k^
	Dyspnea	mMRC^l^
	Fatigue	BFI-K^m^
	Sleep	PSQI-K^n^
	Distress in cancer patient	NCCN DT & PL°
	Depression	PHQ-9^p^
	Cognitive function	K-AD8^q^
	Capability for daily activities	K-IADL^r^

AUDIT^a^, Alcohol Use Disorder Identification Test.

MDA^b^, Mini Dietary Assessment.

IPAQ-SF^c^, International Physical Activity Questionnaire-Short Form.

EMR^d^, Electronic Medical Record.

STS & ESTS definition^e^, Standardized Definitions of Society of Thoracic Surgery & European Society of Thoracic Surgery.

C-D classification^f^, Clavien-Dindo Classification.

PG-SGA-SF^g^, Patient-Generated Subjective Global Assessment Short Form.

EQ-5D-5L^h^, EuroQol 5-Dimensions 5 Levels.

EORTC QLQ-C30^i^, European Organization for Research and Treatment of Cancer Quality of Life Questionnaire-Core 30.

EORTC QLQ-OES18^j^, European Organization for Research and Treatment of Cancer-Esophageal Module 18.

GSRS^k^, Gastrointestinal Symptom Rating Scale.

mMRC^l^, modified Medical Research Council Dyspnea Scale.

BFI-K^m^, Korean version of the Brief Fatigue Inventory.

PSQI-K^n^, Korean version of the Pittsburgh Sleep Quality Index.

NCCN DT & PL°: The National Comprehensive Cancer Network Distress Thermometer and Problem List.

PHQ-9^p^, Patient Health Questionnaire-9.

K-AD8^q^, Korean version of Alzheimer disease 8.

K-IADL^r^, Korean version of Instrumental Activities of Daily Living.

To investigate the relationship between disease characteristics and outcomes, the disease and treatment information domain comprises baseline comorbidities, baseline pulmonary function, esophageal cancer information, cancer treatment information, complications, disease progression, and survival status. Every information within this domain is collected by reviewing electronic medical records (EMR), with some comorbidity data gathered using self-developed questionnaires according to anatomic classification. Information on newly diagnosed disease following treatment is also collected during the patient’s study visits.

The nutritional status domain includes muscle strength and nutritional indices, such as body weight, body mass index (BMI), laboratory results, and overall nutritional evaluation. Muscle strength is measured by averaging the grip strength dynamometer values from both hands. Laboratory results consist of albumin, total protein, hemoglobin, white blood cell, absolute lymphocyte count, absolute neutrophil count, and platelet. Data on each variable is collected at every visit from T0 to the end of the study period. Body weight, BMI, and laboratory results are obtained through EMR review, whereas the nutritional assessment utilizes Patient-Generated Subjective Global Assessment Short Form.

The QoL domain includes overall QoL, QoL in patients with cancer, QoL in patients with esophageal cancer, gastrointestinal symptoms, dyspnea, fatigue, sleep, distress in cancer patient, depression, cognitive function, and capability for daily activities. These variables are used to assess the burden of symptoms and QoL of the patients from various perspectives. QoL is collected at baseline; T0, pre-treatment (T1, if applicable), 6 months post-treatment (T4), and annually from 12 months post-treatment (T5), using PROMs (**Table 2**). Cognitive function is assessed biannually from T5.

### 2.3 Statistical analysis

Based on the assumption that approximately 20% of esophageal cancer survivors experience significant issues related to impaired HRQoL, we estimated that at least 80 patients with substantial unmet needs would be required to develop predictive models and define intervention targets. Considering potential attrition and the need for subgroup analyses, we determined that a total sample size of 600 participants would be appropriate.

We will classify patients into a “risk group” (those experiencing a clinically significant decline in QoL by year 3 post-surgery) and a “non-risk group,” and compare baseline characteristics using t-tests and chi-square tests, as appropriate. Mixed-effects models will then be used to examine longitudinal differences in QoL trajectories between the two groups. To examine longitudinal differences in QoL trajectories between the two groups, we will employ linear mixed-effects models to account for repeated measurements within individuals. In these models, time will be treated as a fixed effect, and participant identifier will be included as a random intercept to model intra-individual correlation. To account for potential heterogeneity across cohorts, such as differences in recruitment period or baseline characteristics, the cohort source will also be modeled as a random effect. This hierarchical structure will allow us to account for clustering without inflating the variance of fixed-effect estimates. Baseline covariates and interaction terms will be included to explore effect modification over time. Variable selection will be informed by existing literature and preliminary univariable analyses, with the final model determined using likelihood ratio tests (significance level set at p < 0.05). Multivariable logistic regression will be conducted to identify predictors of risk group membership, including variables such as baseline nutritional status (BMI, PG-SGA-SF), symptom severity, and psychosocial factors.

Planned subgroup analyses will be stratified by age (<65 vs ≥65), sex, type of surgery (e.g., minimally invasive vs open), and treatment modality (surgery alone vs surgery + neoadjuvant or adjuvant therapy). These strata were chosen based on prior studies showing differential impacts on QoL and recovery patterns among esophageal cancer survivors. Sensitivity analyses stratified by cohort will be conducted to assess the robustness and consistency of findings.

## 3 Results

We expect that > 50% of long-term survivors will be enrolled from existing cohorts, and that newly diagnosed patients will also be recruited. As of June 2025, the median follow-up duration since their primary treatment was 72.8 months for the participants from CATCH-EGO-C (n = 90), 37.9 months for the participants from ASSURE (n = 122), and 22.7 months for the participants from CATCH-EGO-I (n = 84). As the cohorts are still under active follow-up and final data consolidation is pending, loss to follow-up rates have not yet been finalized and are not reported in this protocol paper. For newly enrolled patients (n = 152), follow-up is also ongoing, with a median follow-up duration of 5.2 months from surgery (**Table 3**).

**Table 3:** Baseline characteristics.

Variables	N(%)
Source cohort	
CATCH-EGO-C	90 (20.1)
ASSURE	122 (27.2)
CATCH-EGO-I	84 (18.8)
Newly diagnosed	152 (33.9)
Treatment plan	
Surgery	239 (53.3)
Surgery + preoperative chemoradiotherapy	128 (28.6)
CCRT^a^	81 (18.1)
Clinical stage at diagnosis	
I - II	383 (85.5)
III or above	65 (14.5)
Follow-up period since primary treatment	
< 1 year or awaiting surgery	155 (34.6)
≥ 1 year and < 3 years	131 (29.2)
≥ 3 years and < 5 years	76 (17.0)
≥ 5 years	86 (19.2)

CCRT^a^: Concurrent Chemoradiation Therapy.

## 4 Discussion

### 4.1 Key strategies

This study aims to establish a TRC of patients with esophageal cancer through prospective recruitment from multiple centers. The primary objective is to develop a comprehensive cohort capable of assessing the unmet needs and HRQoL of survivors, and to facilitate timely referral to appropriate interventions when patients are eligible and willing to participate. To achieve this, our TRC employs three key strategies. First, we leverage three existing prospective studies to efficiently identify and enroll long-term survivors. Second, we collect a wide range of data on HRQoL, health behaviors, and environmental factors to enable a multidimensional understanding of survivorship. Third, we collect multiple times within short-term interval points, allowing seamless linkage between the cohort and appropriate trials.

First, we use three existing prospective studies to efficiently identify and enroll long-term survivors. Although esophageal cancer highlights the critical need for long-term research, previous esophageal cancer cohorts have generally enrolled < 200 patients and have faced significant losses to follow-up (20). Given the low incidence and high mortality of the disease, enrolling and retaining patients over time is inherently difficult. To overcome these challenges, we adopted an innovative cohort development strategy to enhance recruitment efficiency and build a robust longitudinal dataset. Specifically, we implemented a dual enrolment approach that includes both newly diagnosed patients and long-term survivors from three existing prospective studies: CATCH-EGO-C, ASSURE, and CATCH-EGO-I. These studies were originally designed to collect baseline HRQoL data, which is often lacking in retrospective or real-world datasets. By leveraging these cohorts, we aim to efficiently recruit long-term survivors within a relatively short timeframe, reducing both time and resource burdens while maintaining high-quality data. Each cohort contributes distinct strengths—CATCH-EGO-C focused on physical activity, ASSURE collected detailed adverse event data, and CATCH-EGO-I incorporated lifelog-based nutritional and physical activity interventions. Despite these differences, all three studies utilized identical patient-reported outcome measures (PROMs) and aligned measurement timepoints, allowing for integration into a single, unified cohort with consistent and comparable QoL data.

Second, we collect a wide range of data to enable a multidimensional understanding of survivorship. Prior qualitative studies have shown that survivors of esophageal cancer report complex challenges in managing their symptoms, diet, psychosocial factors, and social support (21). A focus group study revealed that the HRQoL of patients is heavily influenced by various interconnected factors. For instance, physical symptoms such as diarrhea lead to psychological and social issues, including anxiety and isolation (22), highlighting the need for multidimensional research on QoL in survivors of esophageal cancer. However, many studies used limited measurements, capturing only a narrow scope of patient QoL. For instance, previous studies such as the Prospective Observational Cohort Study of Esophageal-gastric cancer and the Swedish National Registry for Esophageal and Gastric Cancer (NREV) have relied on the European Organization for Research and Treatment of Cancer (EORTC) Quality of Life Questionnaire Core 30 (QLQ-C30) and its esophageal cancer-specific module, QLQ-OG25, to assess QoL in patients with esophageal cancer (10, 23, 24). The EORTC QLQ-C30 is designed to measure cancer-related QoL across five functional domains and various symptom scales, while the QLQ-OG25 focuses on esophageal-specific symptoms such as dysphagia, reflux, and eating difficulties. Although these instruments are valuable, they do not comprehensively capture all aspects of QoL relevant to esophageal cancer survivors. Chronic issues such as altered gastrointestinal function, severe fatigue, dietary restrictions, sleep disturbances, and fear of cancer recurrence—common after esophagectomy—are often underrepresented. To address this gap, our study employs a broader set of QoL assessments that capture long-term survivorship issues in greater detail. In fact, a potential application currently under development involves a structured nutritional intervention program tailored to the unique dietary challenges faced by esophageal cancer survivors. This detailed understanding will facilitate the identification of specific unmet needs and enable the design of tailored interventions or clinical trials, contributing to a more comprehensive approach to survivorship care in esophageal cancer.

Third, our study collects multiple time points, enabling us to track the trajectory of HRQoL from the preoperative phase onwards. This approach also allows seamless linkage between the cohort and appropriate intervention trials. Curative surgery remains the standard primary treatment for esophageal cancer, but it leads to a permanent decline in gastric function, resulting in significant physiological changes. Therefore, substantial differences in patient conditions before and after surgery are well recognized (15, 25). Despite this, many previous studies such as Swedish cohort study have not included preoperative QoL assessments (10, 24, 26–29). Consequently, these studies such as NREV cohort could only report postoperative QoL trends or relied on alternative methods for comparison (24). Additionally, previous studies such as Sweden cohort study have assessed patient QoL at relatively long intervals. QoL assessments in prior research typically occurred at 6 months, 3 years, and 5 years post-surgery, with intervals of at least 5 years, depending on the study objectives (26, 28). To overcome these limitations, we collect data at shorter intervals during annual assessments. Also, by measuring QoL in its most fluctuant and vulnerable period; immediately post-surgery; we can gain valuable insights into short-term changes that may influence long-term outcomes. We believe that gathering comparable and periodic data provides a clearer understanding of the overall trends in the QoL of survivors and offers new insights into the relationship between the postoperative period and long-term QoL outcomes.

### 4.2 Limitations

Despite these strengths, our study has some limitations. First, the predominance of Korean participants may limit the generalizability of our findings. There are epidemiological differences in esophageal cancer between Asian and Western populations; while adenocarcinoma accounts for > 60% of esophageal cancer cases in the West, squamous cell carcinoma is more common in the East (30). This distinction influences treatment approaches, with squamous cell carcinoma often requiring total esophagectomy, leading to significant and permanent physical changes that profoundly affect QoL (31). Therefore, our findings may not be generalizable to populations with different epidemiological profiles. We aim to mitigate this limitation by conducting this study as a multicenter study to include patients from a broader range of clinical settings, which may help capture some variability in patient characteristics. However, future expansion of the TRC to multinational settings is currently under discussion. In particular, we are exploring collaborations across East Asia, where squamous cell carcinoma predominates, and considering adaptations of the PROM framework to accommodate Western cohorts primarily affected by adenocarcinoma. This would allow for cross-cultural validation and broader applicability of our findings.

### 4.3 Implications

This study presents a well-designed TRC of survivors of esophageal cancer. Although constructing a large longitudinal cohort of this population poses challenges, we have implemented a distinct strategy to overcome these limitations. We anticipate that our cohort will provide valuable data to generate new insights into the QoL of long-term survivors of esophageal cancer and serve as a platform for prompt connections toward proper interventions. While this manuscript describes the initial design and infrastructure of the TRC, the cohort is actively ongoing, with longitudinal follow-up proceeding according to protocol. Once complete, the cohort is likely to be widely cited as a methodological and data-rich reference point in the field of esophageal cancer survivorship.

## Data Availability

The raw data supporting the conclusions of this article will be made available by the authors, without undue reservation.
